# TrackdAT, an acoustic telemetry metadata dataset to support aquatic animal tracking research

**DOI:** 10.1038/s41597-024-02969-y

**Published:** 2024-01-30

**Authors:** Jordan K. Matley, Natalie V. Klinard, Ana Barbosa Martins, Arun Oakley-Cogan, Charlie Huveneers, Christopher S. Vandergoot, Aaron T. Fisk

**Affiliations:** 1 College of Science and Engineering, Flinders University, Bedford Park, SA 5042 Australia; 2 Department of Biology, Dalhousie University, Halifax, NS B3H 4R2 Canada; 3 Department of Fisheries and Wildlife, Michigan State University, East Lansing, MI 48824 USA; 4 Great Lakes Institute for Environment Research, University of Windsor, Windsor, ON N9B 3P4 Canada

**Keywords:** Ecology, Scientific community

## Abstract

Data on the movement and space use of aquatic animals are crucial to understand complex interactions among biotic and abiotic components of ecosystems and facilitate effective conservation and management. Acoustic telemetry (AT) is a leading method for studying the movement ecology of aquatic animals worldwide, yet the ability to efficiently access study information from AT research is currently lacking, limiting advancements in its application. Here, we describe TrackdAT, an open-source metadata dataset where AT research parameters are catalogued to provide scientists, managers, and other stakeholders with the ability to efficiently identify and evaluate existing peer-reviewed research. Extracted metadata encompasses key information about biological and technical aspects of research, providing a comprehensive summary of existing AT research. TrackdAT currently hosts information from 2,412 journal articles published from 1969 to 2022 spanning 614 species and 380,289 tagged animals. TrackdAT has the potential to enable regional and global mobilization of knowledge, increased opportunities for collaboration, greater stakeholder engagement, and optimization of future ecological research.

## Background & Summary

Investigating the movements of aquatic animals in relation to biotic and abiotic drivers generates key information about ecological interactions in diverse ecosystems^1^. Knowledge of movement ecology is also critical to facilitate management that effectively protects or sustainably exploits aquatic organisms^2^. For example, understanding migratory routes, foraging areas, and reproductive sites of animals throughout different stages of their life history can help to develop evidence-based conservation strategies or fishery guidelines^3,4^. Acoustic telemetry has become a leading method to study the movement ecology of fishes (including sharks and rays) and other animals in rivers, lakes, estuaries, and oceans from polar regions to the tropics due to its ability to track aquatic animals autonomously in their natural environment at increasingly relevant spatial and temporal scales^5^. Acoustic telemetry (AT) is a technology that uses acoustic signals to characterize the presence, movements, and behaviour of animals in marine and freshwater habitats. A small electronic transmitter is implanted internally or attached externally to the animal. The transmitter emits an acoustic signal that can be detected by a hydrophone or receiver (typically moored on the bottom but can also be deployed on mobile platforms) when the tagged animal swims nearby, permitting continuous, long-term monitoring up to and exceeding 10-year periods. The application of acoustic telemetry to study aquatic animal movements has grown exponentially since its inception with more than 2400 peer-reviewed journal articles published between 1969 and 2022 – a value that is projected to increase >250% over the next 10 years^5^. More than a quarter million aquatic animals, primarily fishes, have been tracked globally during this 50**-**year period. Researchers are increasingly using AT to identify species interactions, critical habitats, timing and spatial extent of movements, ontogenetic shifts, energetic demands, spawning behaviour, and physiological scope associated with movements, ultimately broadening our ecological knowledge of aquatic animals small and large. Furthermore, there are increasing appeals to use AT to bridge the common gap between science and policy relating to pertinent human-environmental issues^2,5,6^. These efforts are being spurred by global initiatives, such as the United Nations Decade of Ocean Sciences for Sustainable Development^7^ and Global Ocean Observation Systems^8^, to increase ocean monitoring, data storage and sharing, and multifaceted collaborations.

The extent of AT research globally has led to an incredible amount of data accumulation that is increasingly being stored among different regional AT collaborative networks. For example, the Canada-based Ocean Tracking Network (OTN) houses AT data (i.e., receiver, transmitter, and detection data) across hundreds of different (and often independent) projects globally. Collaborative AT networks such as OTN have been established throughout the world and include (but are not limited to) the Great Lakes Acoustic Telemetry Observation System (GLATOS; USA), the European Tracking Network (ETN; Europe), Florida Atlantic Coast Telemetry Network (FACT; USA), Integrated Tracking of Aquatic Animals in the Gulf of Mexico (iTAG; USA), Integrated Marine Observing System (IMOS; Australia), and Acoustic Tracking Array Platform (ATAP; South Africa). The goals of these networks vary, but generally aim to increase collaboration and optimize research output through data and equipment sharing^9–11^. While there are numerous AT networks each hosting millions of tracking datapoints, many act independently of each other with spatially segregated purviews. Additionally, they are based on user-volunteered input and not necessarily output (e.g., focus is on data going in, not citable end-products), and do not offer researchers outside of these projects the ability to efficiently explore study metadata (e.g., species tagged, sample size, location, life stage). While there are certainly exceptions to some of the above (e.g., IMOS has a metadata non-proprietary protocol; animaltracking.aodn.org.au/about), there remains discontinuity of data access from a global perspective. Access to standardized global AT metadata is valuable because it can serve to evaluate current research and output, while broadening the scope of and informing future work.

The main objective of this project is to provide scientists, managers, and other stakeholders with the ability to efficiently identify and evaluate existing peer-reviewed AT research by creating an open-source online dataset where AT research parameters are stored, catalogued, and shared. The project aims to optimize future movement ecology research by providing the tools that will lead to regional and global mobilization of knowledge, increased opportunities for collaboration, and greater stakeholder engagement, among other benefits. In this Data Descriptor, we introduce TrackdAT, a dataset compiled of AT study metadata derived from relevant peer-reviewed primary journal articles. TrackdAT enables open access to all published AT research in one central location, creating a multitude of opportunities to broaden the field of movement ecology and application of AT throughout the world. The project will directly contribute to the generation, dissemination, and application of scientific and technological knowledge related to data collection, synthetic reviews, resource management, conservation, aquatic monitoring, and science communication, among others, to support evidence-based decision-making for marine and freshwater ecosystems.

## Methods

**Fig. 1:**
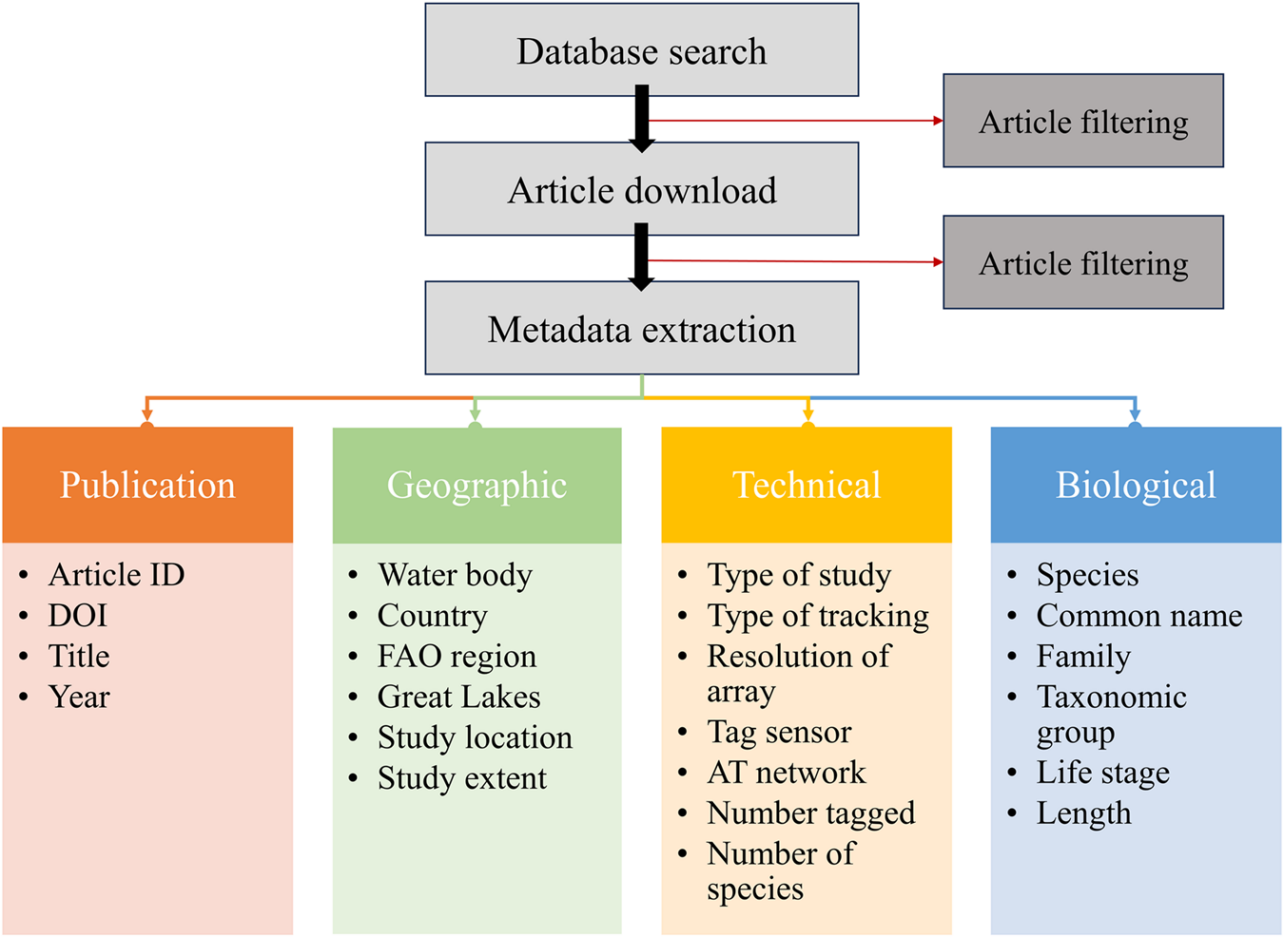
General acoustic telemetry (AT) metadata extraction process. The coloured boxes represent the specific metadata fields that were extracted from each AT article. See *Methods* for specific information about the metadata extraction process.

The TrackdAT dataset is composed of metadata fields extracted directly from peer-reviewed primary AT research articles. Metadata collected from each publication were grouped in four overarching categories (Fig. 1): publication (i.e., article information), geographic (i.e., spatial information), technical (i.e., specialized AT information), and biological (i.e., species-related information), providing a comprehensive summary of each article. See *Data Records* section and Supplementary Table 1 for description of each metadata field extracted. Acoustic telemetry research articles were identified using a multi-criteria search syntax in Web of Science for all years up to and including 2022 (i.e., “Acoustic telemetry” OR “Acoustic tracking” OR “Passive telemetry” OR “Acoustic transmit*” OR “Acoustic receiver*” OR “Acoustic tag*” OR “Ultrasonic tracking” OR “Ultrasonic telemetry” OR “Fish track*”). Symposium articles were not included. In TrackdAT, we consider AT research to refer specifically to the measurement of spatial data although it otherwise may also encompass physiological data transmitted via sound (i.e., studies that only used AT to record physiological data were not included). A total of 3,807 articles matched the search criteria. Titles were scanned and articles that were clearly unaffiliated with AT were removed (1,176 articles removed). The remaining articles were downloaded and abstracts were scanned to ensure articles were again appropriate (185 further articles removed). If an AT article could not be accessed, it was requested from the corresponding author (34 articles remained inaccessible). A total of 2,412 peer-reviewed primary journal articles were retained for the final dataset. Each AT article was examined by one of three team members (hereafter referred to as administrators). Administrators read the abstract and methods of each article to extract predetermined metadata fields. The *Redact* tool in Adobe Acrobat Pro DC (v.2020.006.20042) was also used to help identify commonly used terms associated with the metadata fields.

## Data Records

TrackdAT can be accessed online at www.trackdAT.org or independently within *figshare*^12^. The main dataset is structured as a downloadable (to .csv format) data record. See Table 1 for a description of each available metadata field. Each metadata field can also be filtered (and downloaded), as well as explored through various interactive tools (e.g., maps, summary plots, species detail pages) at www.trackdAT.org.

**Table 1:** Brief description of each metadata field extracted from peer-reviewed journal articles pertaining to acoustic telemetry (AT) studies. See Supplementary Table 1 for detailed description of each metadata field. Italicized terms in the 'Metadata fields' column are grouped fields that can be distinguished in the 'Brief description' column. Italicized terms in the 'Brief description' column identify specific metadata category options.

Metadata category	Metadata fields	Brief description
Publication	Article_ID	Unique identification of each article (internal use).
	*Article info*	Consists of three columns pertaining to article information: ‘DOI’, ‘Title’, and ‘Year’.
Geographic	Water_body	The body of water of the study, options include: *marine*, *freshwater*, and *laboratory*.
	Country	The country in which the study was conducted.
	FAO_region	The FAO Major Fishing Area.
	Great_Lakes	Identifies whether the study was conducted within the Great Lakes basin.
	*Study location*	Consists of two columns pertaining to the study location: ‘Longitude’ and ‘Latitude’.
	Study_extent	Identifies whether the study was conducted in an area larger than 100 km linear distance.
Technical	Type_of_AT_study	Category of the study, options include: *Ecology*, *Survival*, *Tagging effects*, *Methodology, Range testing*, *Technology*, and *Review*
	Type_of_tracking	The broad method of tracking, options include: *stationary*, *mobile*, *both*, or *neither*.
	Resolution_of_array	Identifies whether a high-resolution tracking system was used.
	Tag_sensor	Identifies if any tag sensor was used, options include: *pressure/depth*, *temperature*, *acceleration*, *feeding*, *digestion/predation*, *illumination*, *speed*, *salinity*, or *none*.
	AT_network	Identifies whether the study was associated with an AT collaborative network.
	Number_tagged	Quantifies the total number of individuals that were tagged in the study.
	Number_of_species	Quantifies the number of unique species that were tagged in the study
Biological	Species	Scientific name of the species tracked using Fishbase^14^ or Sealifebase^22^.
	Common_name	Common name used in the article to identify species.
	Species_family	Family of the species that were tracked in the study.
	Broad_taxonomic_group	Colloquial grouping of the species tracked in the study, such as *sharks/rays* and *fish*.
	Life_stage	Identifies the state of maturity of the study animals.
	*Animal length*	Consists of three columns pertaining to the size of tagged study animals: ‘Minimum_length’, ‘Maximum_length’, and ‘Mean_length’.

**Fig. 2:**
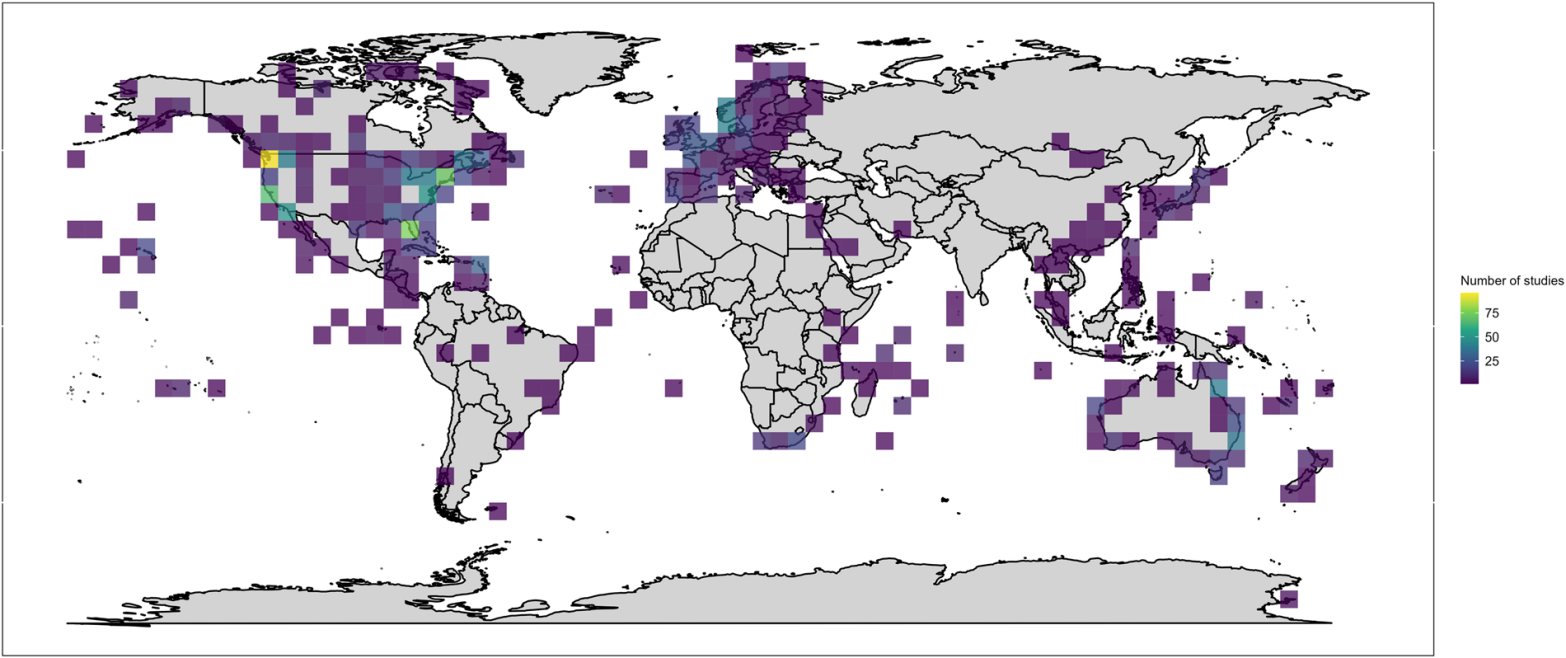
Geographic locations of studies. Each grid (5° × 5°) contains the sum of unique articles published between 1969 and 2022 (n = 2,062). Note that locations were only assigned to *Ecology, Survival, Tagging effects*, and *Range testing* study types.

**Fig. 3:**
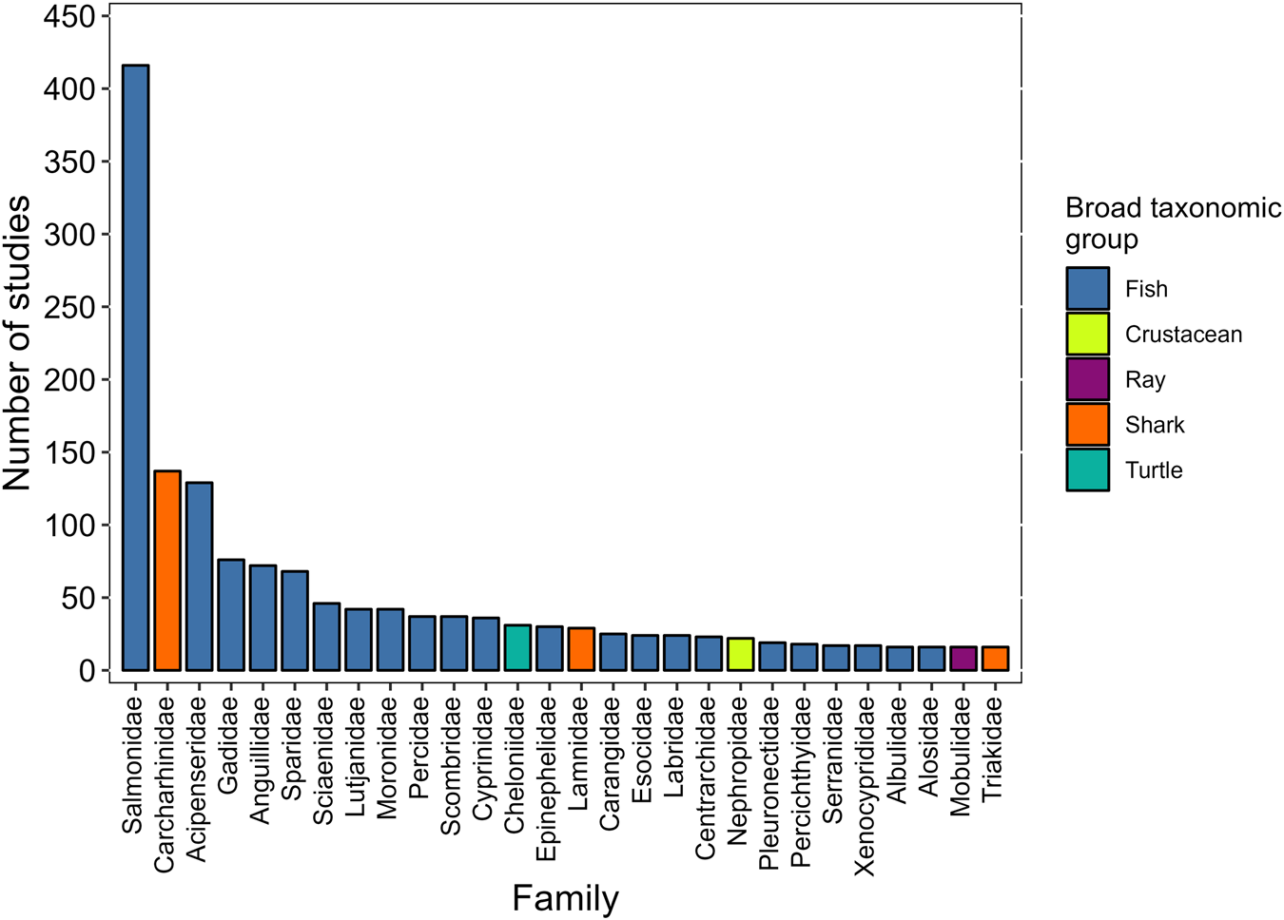
Family of study species. The total number of articles distinguished by the family of study species and categorized into broad taxonomic groups. Families displayed are subset to those that consisted of more than 15 studies.

**Fig. 4:**
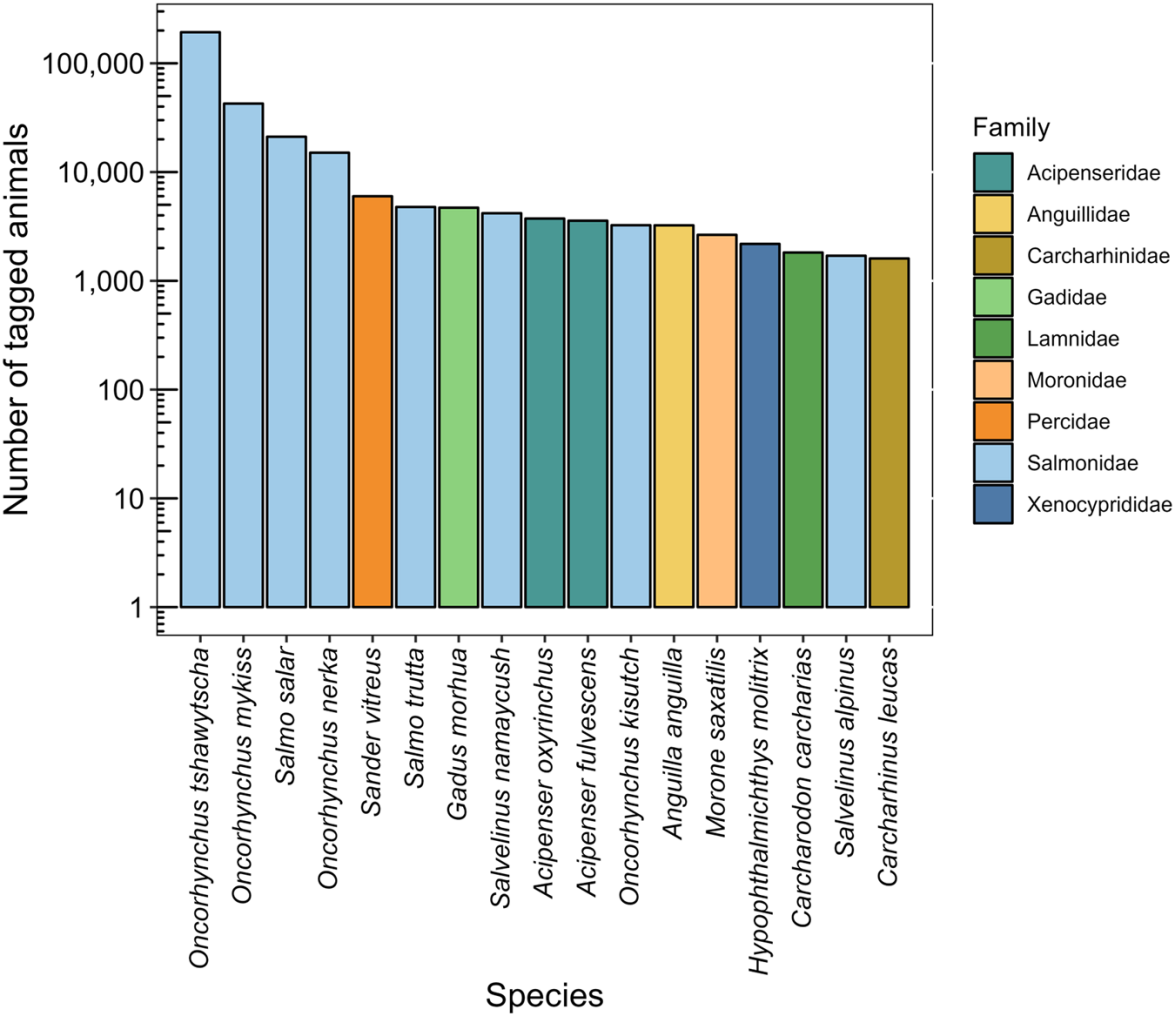
Number of tagged animals by study species. The total number of tagged animals grouped by study species and categorized by family. Species displayed are subset to those that consisted of more than 1,500 tagged individuals.

At present, the datset contains 2,412 peer-reviewed primary journal articles published between 1969 and 2022 (Fig. 2). Studies have been conducted in freshwater, estuarine, and marine environments associated with 73 unique countries. A total of 614 species belonging to 175 families have been studied with Osteichthyes (i.e., bony fish) and Chondrichthyes (i.e., sharks and rays) being the broad taxonomic groups most studied (Fig. 3), although many other animals (e.g., sea turtles, crustaceans, mammals) have also been tracked with AT albeit with less regularity. The number of animals tracked between 1969 and 2022, estimated only from *ecology*, *survival*, and *tagging effects* studies, is 380,289 (Fig. 4). The online database is supported by a dedicated group of administrators and will be updated (i.e., new articles added) at www.trackdAT.org website at weekly or monthly intervals, whereas the stored dataset in *figshare* will be updated annually (i.e., the current peer-reviewed dataset is Version 1).

## Technical Validation

Metadata, as described in Table 1, has been extracted directly from peer-reviewed primary journals. Potential sources of error can be attributed to two categories: missed articles and erroneous data extraction.

### Missed articles

Missed articles refers to not incorporating relevant articles into the dataset. The search criteria used to identify AT articles was broad, typically requiring removal of unsuitable articles (e.g., hydroacoustic studies) rather than omitting suitable ones. Missed articles also pertain to studies that were not accessible under copyright regulations or published in journals that are not included in Web of Science (e.g., regional journals). Our preliminary article identification stage compared other search databases with Web of Science (i.e., Scopus, Google Scholar) and showed that only a limited number of articles were missed, primarily from specific journals; therefore, these journals were also accessed independently throughout the article selection process. Furthermore, we recognize that not all research and output is confined to peer-reviewed primary journals (e.g., government reports); but these were outside the scope of the dataset in its current capacity. Finally, our search was limited to research published in English and may not include studies reported in other languages. An important component of TrackdAT is the ability for users to notify TrackdAT administrators and suggest missed articles to include. Users have the option of filling in metadata fields themselves or requesting an administrator to do so. Regardless of the approach, each new input is validated by a senior administrator, and adjusted if needed.

### Erroneous data extraction

All data extraction was completed by one of three senior administrators who have at least five years of practical experience using AT. Administrators underwent collaborative consultation and training prior to developing data extraction and organization procedures. Regular meetings and discussions were held to ensure consistency when populating the dataset. Conferral among the other administrators was completed when required, and double assignments of the same articles were performed regularly to evaluate precision between administrators. These tests resulted in >95% precision rates. Like above, users can notify administrators about potential errors in the dataset. These suggestions are validated by a senior administrator and adjusted if appropriate.

## Usage Notes

TrackdAT enables global access to AT metadata from all available peer-reviewed research. The ability to concisely and rapidly view, download, and visualize study information creates significant opportunities to develop and strengthen the application of AT in the field of movement ecology. A few examples of how TrackdAT can be used include:

1. Conduct synthetic or review studies using filterable fields (e.g., horizon scans, geographic areas, species, types of studies)^5^
2. Develop collaborations or improve networking by identifying researchers conducting research of interest^13^
3. Identify current or ongoing gaps in research (e.g., geographic areas, species, research topics)^5^
4. A starting place for new students to learn about AT and movement ecology or to highlight unknown research for more established researchers
5. A citable resource to confirm topical research has been conducted (e.g., “the TrackdAT dataset was filtered to identify previous articles that tracked the movements of Atlantic salmon”)
6. Evaluate performance and identify areas of expertise within the AT community (e.g., authors with multiple research output on specific topics)
7. Expand the current scope of dataset by connecting outside of TrackdAT (e.g., Fishbase^14^, Sharkipedia^15^)
8. Evidence to funders of research output or rationalization for funding/grant/ethics applications
9. A readily accessible record of recent developments in methodology, technology, and novel analyses to support the rapidly growing field of AT and technological advancements that produce increasingly large and complex datasets^16^
10. Seek information about methods used, animal welfare, and sample size for ethics applications

The list above is not exhaustive – TrackdAT has significant opportunity to help inform researchers and managers in many ways, especially as it continues to be developed. Various output using the precursor dataset to TrackdAT already exist, including an evaluation of mortality in AT research^17^, a summary of global trends and application of AT to support management^5^, an assemblage of complementary methods to bolster AT application^18^, and a synthesis of the growing use of acoustic accelerometer transmitters in aquatic science^19^. By sharing this dataset publicly, we hope to create new opportunities to collaborate and consolidate research, and ensure that future work is inclusive, efficient, ethical, cost-effective, and impactful. We also plan to facilitate engagement and outreach among different stakeholders (e.g., Great Lakes Fishery Commission meetings), as well as the public to help bridge knowledge-action gaps relating to aquatic animal movements^20^. TrackdAT has been created to follow FAIR Data Principles^21^ by being findable, accessible, interoperable, and reusable.

### Supplementary information

Supplementary Material (PDF)

Supplementary Material (xlsx)
