# Supplementary material for: TrackdAT, an acoustic telemetry metadata dataset to support aquatic animal tracking research
Source: Sci Data. 2024 Jan 30;11:143. doi: 10.1038/s41597-024-02969-y (PMC10828395; doi:10.1038/s41597-024-02969-y)
Supplement: Supplementary file 1 — Supplementary Material (PDF) [file 41597_2024_2969_MOESM1_ESM.pdf]

Supplementary Material

TrackdAT, an acoustic telemetry metadata dataset to support aquatic animal tracking research

Jordan K Matley[1], Natalie V Klinard[2], Ana Barbosa Martins[2], Arun Oakley-Cogan[2], Charlie Huveneers[1], Christopher S Vandergoot[3], Aaron T Fisk[4]

1. College of Science and Engineering, Flinders University, Bedford Park, SA, Australia 5042
2. Department of Biology, Dalhousie University, Halifax, NS, Canada B3H 4R2
3. Department of Fisheries and Wildlife, Michigan State University, East Lansing, MI, USA, 48824
4. Great Lakes Institute for Environment Research, University of Windsor, Windsor, ON, Canada, N9B 3P4

corresponding author: Jordan Matley (jordan.matley@flinders.edu.au)

Supplementary Table 1: Detailed description of each metadata field extracted from peer-reviewed journal articles pertaining to acoustic telemetry (AT) studies.

Metadata category	Metadata field	Description
Publication	Article_ID	Unique identification of each article (internal use).
Publication	*Article info*	Consists of three columns pertaining to article/publication information: 'DOI', 'Title', and 'Year'.
Geographic	Water_body	The body of water in which the study was conducted. Options include: *marine*, *freshwater*, *marine/freshwater* (i.e., estuarine or both marine and freshwater), or *laboratory* (i.e., controlled laboratory or small pond setting).
Geographic	Country	The country in which the study was conducted (e.g., location of acoustic array). If conducted outside the Exclusive Economic Zone, an 'International' designation was used.
Geographic	FAO_region	The FAO Major Fishing Area (fao.org/fishery/en/area/search) where the study was conducted. If a study was conducted in both freshwater and marine (or estuarine) environments, the applicable marine FAO area is used. Otherwise, if a study was conducted in more than one FAO region, the region where the majority of work was done is used.
Geographic	Great_Lakes	Identifies whether the study was conducted within the Great Lakes basin (glc.org/greatlakesgis). Options include: *yes* or *no*.
Geographic	*Study location*	Consists of two columns pertaining to the study location: 'Longitude' and 'Latitude' (measured in decimal degrees). Coordinates provided in text are used unless they fell on land upon verification. If coordinates were not provided (or they fell on land), the location of study is estimated in Google Earth based on information provided in the article. When estimating locations, the approximate center of the study area (e.g., receiver array) is used unless the areas were distinctively grouped at unique locations, at which point the central or more focal area is used.
Geographic	Study_extent	Identifies whether the study was conducted in an area larger than 100 km linear distance (i.e., maximum linear distance between receivers).
Technical	Type_of_AT_study	Category of the study that best matched one of the following definitions:<br>*Ecology*: exploration of the movement and behaviour of tagged aquatic animals *in situ*<br>*Survival*: estimation of mortality or survival based on interpretation of movement, sensor, or recapture data<br>*Tagging effects*: investigation of the behavioural, health, or other impacts associated with

		the process of inserting or attaching tags into or on an animal<br>*Methodology*: development or application of a novel method or analysis to facilitate more accurate or efficient interpretation of AT data.<br>*Range testing*: quantification of the factors (e.g., distance, temperature, noise) that influence the detectability of acoustic transmissions in aquatic environments<br>*Technology*: development or evaluation of novel AT equipment or technology<br>*Review*: synthesis or summary of information from previous AT research that adheres to specific themes or topics, including opinion-based articles or data descriptions<br>*Note, if more than one type of study was relevant to an article, the focal type is selected based on stated objectives. Also, metadata categories were not extracted for some study types due to illogical nature of them (e.g., review studies were not assigned a location).
Technical	Type_of_tracking	The broad method of tracking that was used in the study. Options include: *stationary* (i.e., the use of stationary receivers to passively track animals), *mobile* (i.e., the use of mobile receivers either actively or passively tracking animals), *both* (i.e., a combination of stationary and mobile tracking), or *neither* (e.g., in a tagging effects study).
Technical	Resolution_of_array	Identifies whether a high resolution tracking system (i.e., using speed of sound to triangulate detections and estimate location with high degree of accuracy) was used. Only stationary tracking methods are considered capable of high resolution. Options include: *yes* or *no*.
Technical	Tag_sensor	Identifies whether a sensor was built into the acoustic transmitter enabling environmental or biological measurements separate from simply location or presence/absence data. Options include: *pressure/depth*, *temperature*, *acceleration*, *feeding*, *digestion/predation*, *illumination*, *speed*, *salinity*, or a combination thereof. This list is updated as new sensors are used.
Technical	AT_network	Identifies whether the study was associated with or used equipment procured by an existing AT collaborative network. Only if the article stated the network affiliation (within article or acknowledgements) is it included (i.e., there may be an unstated association). Options include: *Atlantic Cooperative Telemetry (ACT) Network, Florida Atlantic Coast Telemetry (FACT) Network, Integrated Marine Observing System (IMOS), Integrated Tracking of Aquatic Animals in the Gulf of Mexico (iTAG), Ocean Tracking Network (OTN), Acoustic Tracking Array Platform (ATAP), California Fish Tracking Consortium (CFTC), Pacific Ocean Shelf Tracking (POST), European Tracking Network (ETN), Great Lakes Acoustic Telemetry Observation System (GLATOS), MigraMar, Mid-Atlantic Acoustic Telemetry Observation System (MATOS), US Caribbean Acoustic Network (USCAN)*, or a combination thereof. This list is updated as new networks are used.
Technical	Number_tagged	Quantifies the total number of individuals that were tagged in the study.
Technical	Number_of_species	Quantifies the number of unique species that were tagged in the study
Biological	Species	Scientific name of the species tracked using taxonomic nomenclature from Fishbase[1] or Sealifebase[2].
Biological	Common_name	Common name used in the article to identify species. If not provided, it is taken from Fishbase[1] or Sealifebase[2]. Note common names can vary; it is suggested that species name is used.
Biological	Species_family	Family of the species that were tracked in the study using taxonomic nomenclature from Fishbase[1] or Sealifebase[2].
Biological	Broad_taxonomic_group	Colloquial grouping of the species tracked in the study. For example, Chondrichthyes are referred to here as *sharks/rays* and Osteichthyes as *fish*. Other common groupings include: *crab, lobster, turtle, seal,* and *snake* (this is not a complete list). Taxonomic accuracy is not prioritized in this field as it aims to be accessible to a wide audience.
Biological	Life_stage	Identifies the broad state of maturity of the study animals based on information provided within the article. If state of maturity was not provided, it is estimated based on the size range of animals tagged using Fishbase[1] or Sealifebase[2]. If insufficient information is available to estimate maturity, the field is left blank. Options include: *juvenile*, *adult*, or

		both. Maturity of salmonids was based on definitions provided by Mobley et al.[3].
Biological	*Animal length*	Consists of three columns pertaining to the size of tagged study animals: 'Minimum_length', 'Maximum_length', and 'Mean_length' (measured in cm). If multiple measurements were given (e.g., fork length and total length), the larger metric is used. If the mean length was not provided in a study or consisted of a table with raw values for >10 individuals, it is estimated as the mean of the minimum and maximum lengths. If median was provided in a study instead of mean, the median is used in place of the mean.

References (numbering specific to Supplementary material and independent of main document)

1. Froese, R. & Pauly, D. FishBase. World Wide Web electronic publication. www.fishbase.org (2023).
2. Palomares, M.L.D. & Pauly, D. SeaLifeBase. World Wide Web electronic publication. www.sealifebase.org (2023).
3. Mobley, K. B. *et al.* Maturation in Atlantic salmon (*Salmo salar*, Salmonidae): a synthesis of ecological, genetic, and molecular processes. *Rev. Fish Biol. Fish.* **31**, 523-571 (2021).
